# Corneal densitometry measurements comparison between anterior segment OCT and scheimpflug imaging

**DOI:** 10.1007/s10792-024-03309-0

**Published:** 2024-09-25

**Authors:** Enrico Lupardi, Antonio Moramarco, Federico Cassini, Simone Febbraro, Giacomo Savini, Luigi Fontana

**Affiliations:** 1 IRCCS Azienda Ospedaliero-Universitaria di Bologna, Bologna, Italy; 2 DIMEC, Ophthalmology Unit, Alma Mater Studiorum Università di Bologna IT, Bologna, Italy; 3 IRCCS Bietti Foundation, Rome, Italy

**Keywords:** Corneal densitometry, Corneal transparency, Corneal opacity, AS-OCT, Scheimpflug Imaging

## Abstract

**Purpose:**

To evaluate and compare the repeatability of corneal densitometry (CD) measurements obtained using both an anterior-segment optical coherence tomography (AS-OCT) device and a Scheimpflug camera system, while also assessing the level of agreement. The study also sought to investigate the correlation of CD with age, gender, and central corneal thickness (CCT) in normal eyes.

**Methods:**

CD measurements were obtained using the Casia 2 and the Pentacam AXL Wave. Data were collected on Total Corneal Densitometry and 4 concentric corneal annular areas, these are referred to as zone 1, denoting the central area, through to zone 4, designating the outermost peripheral region. Repeatability was assessed using intra-session test–retest variability, coefficient of variation (CoV), and intraclass correlation coefficient (ICC). The agreement was evaluated using Bland–Altman plots. Correlation analysis was performed between CD, age, gender, and CCT.

**Results:**

The study included 96 healthy volunteers. The Casia 2 demonstrated high repeatability with ICC values exceeding 0.9 in all the corneal zones and lower CoV values compared to the Pentacam AXL Wave (ranging from 1.07% to 2.25% for Casia 2 and from 1.91% to 6.89% for Pentacam).95% LoA were within ± 2 standard deviation from the average mean except from zone 1 (± 2.42).However, the measurements showed a consistent bias among all the corneal zones. CD values were positively correlated with age, except for zone 1 with the Pentacam (*p* = 0.083).

**Conclusions:**

The findings suggest that the Casia 2 can be a reliable tool for assessing corneal transparency in healthy individuals, however its measurements are not interchangeable with those provided by the Pentacam. The AS-OCT device may be more sensitive in detecting subtle age-related changes in CD within the central zone.

**Supplementary Information:**

The online version contains supplementary material available at 10.1007/s10792-024-03309-0.

## Background

Loss of corneal transparency may result from various causes such as infections, corneal dystrophies, injuries, and degeneration, which may disrupt the cornea’s anatomy and alter its metabolism. Corneal opacification causes increased light scattering and hinders light transmission, resulting in loss of vision [1]. Clinical evaluation of corneal transparency using slit lamp biomicroscopy allows a subjective qualitative or semiquantitative assessment of opacity density and of the impaired visibility of iris details; however, the reproducibility and repeatability of this evaluation among different examiners are limited [2, 3]. Recent advancements in corneal imaging techniques have enabled objective evaluation of corneal transparency through the measurement of corneal light backscattering [4, 5]. This measurement, commonly referred to as corneal densitometry (CD), can be used as an indicator of corneal health [6]. Scheimpflug cameras, such as the Pentacam (Oculus Optikgeraete GmbH; Wetzlar, Germany), have gained popularity as tools for assessing CD in healthy human eyes [4, 7–10], and in various diseases, including diabetes mellitus [11], corneal infections [6], keratoconus [12], Fuchs endothelial dystrophy [13], refractive surgery [14, 15], and keratoplasty [16, 17].

Anterior-segment optical coherence tomography (AS-OCT) is another technology able to provide a quantitative CD analysis [18–21], but has rarely been used for this purpose due to the lack of automated and user-friendly software for CD assessment. The latest software release of the Casia 2 (Tomey Corp., Nagoya, Japan) is the first to include automated CD measurement. Using a 1310 nm wavelength, this swept-source AS-OCT device can penetrate tissues more deeply than the 420 nm blue-LED light emission of the Scheimpflug camera and, due to this ability, may prove helpful when measuring corneal opacity. Nevertheless, due to its recent introduction, there is a lack of studies on the repeatability of Casia 2 AS-OCT measurements and only one assessing their agreement with those provided by the Pentacam AXL Wave [22]. This study was designed to assess the repeatability of CD measurements by the Casia 2 and to compare them to those provided by the Pentacam AXL Wave Scheimpflug camera system and evaluate the correlation of the CD measurements with age and gender and central corneal thickness (CCT) in normal eyes.

## Methods

This was a prospective study conducted on healthy volunteers. During the enrollment visit, adetailed ophthalmic history was obtained, and a complete eye examination was performed, including best corrected visual acuity assessment, tear break-up time, intraocular pressure measurement by Goldmann applanation tonometry, slit lamp biomicroscopy, and fundus examination. The exclusion criteria included a refractive error greater than or equal to -3 and + 3 diopters (D), a best corrected visual acuity lower than 20/20 (Snellen), clinical evidence or ocular history of any corneal disease, tear break up time of more than 10 s, no history of dry eye disease or relevant related symptoms, ocular surface or anterior segment disease, previous history of corneal trauma, ocular surgery, and contact lens use during the last month prior to the examination. After the first visit, if the patient was deemed eligible for enrollment, a subsequent appointment was scheduled on a different day to perform the densitometry measurements. One eye of each subject was randomly selected for the analysis. The study was performed in accordance with the ethical standards of the Declaration of Helsinki and approved by the hospital ethics committee (protocol number 0002793) of the IRCCS Azienda Ospedaliero-Universitaria of Bologna, Policlinico Sant’Orsola, Italy. Patients were informed about the potential risks and benefits of the procedure, and written informed consent was obtained from all participants.

### Instruments

The Casia2 (software version: 50.6A.02) is a swept-source anterior segment optical coherence tomography (AS-OCT) device that uses a 1310 nm wavelength laser source with a scan rate of 50,000 A-scans per second and a scan depth of 13 mm to obtain images with an axial and transverse resolution of 10 and 30 µm, respectively. The radial scan reaches a diameter of 16 mm, while the raster is a 12 × 12 mm square. To measure CD, the “corneal map” scan type was performed, it is a radial scan method with a scan range of 16 mm, in total, 16 different radial scans are performed.

The Pentacam AXL Wave (software version: 1.27r12) is a high-resolution (1.45 megapixel) rotating Scheimpflug camera system for the analysis of anterior segment parameters. It takes up to 100 images in two seconds using a 420 nm wavelength blue LED light source. To measure CD, the 3D Scan protocol with the “25 pictures” option was performed. This method obtains 25 standard quality pictures of the anterior chamber.

### Procedures

Instruments calibration was carried out before the beginning the measurement session. All patients underwent examination using the two devices on the same day, in a darkened room, in random order, and without pupil dilation. No eye drops were instilled before the examination and subjects were asked to blink before each image acquisition. The automatic acquisition modality was selected in both instruments to decrease the human effect on the measurements. The same operator successfully acquired three repeated measurements with each instrument to assess repeatability. After each acquisition, volunteers were asked to move away from the machine and placed back in position for a new measurement after a few seconds. With both instruments, scans were repeated when an alert signal revealed an insufficient measurement quality.

The process of obtaining measurements did not encounter difficulties, all the subjects were able to undergone three consecutive procedures with both instruments without experiencing discomfort or the need to take prolonged pauses. The Casia 2 was faster with a 0.6 s time per exam against 1 s for the Pentacam AXL Wave.

### Measured parameters

**Fig. 1:**
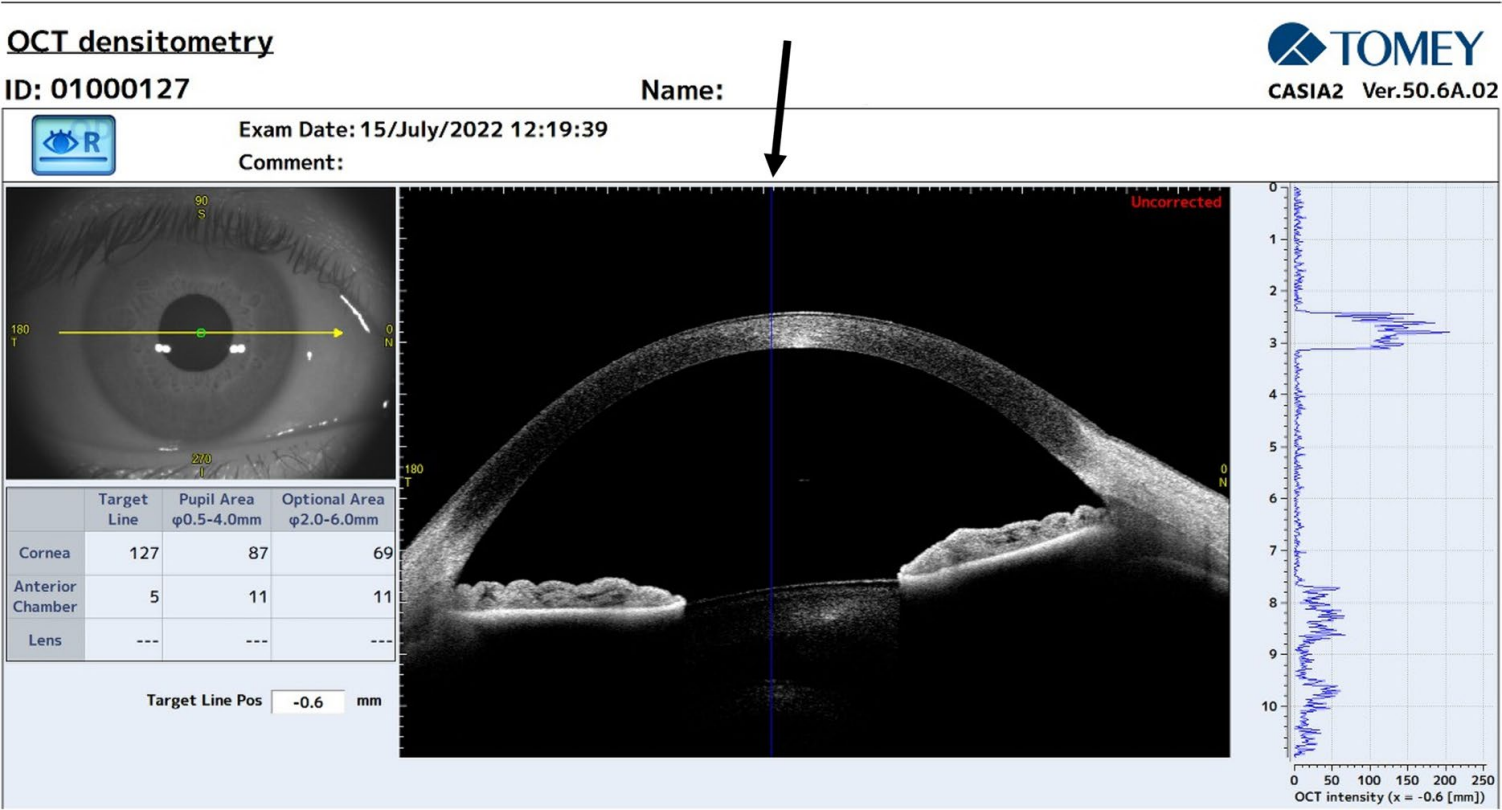
Image illustrating the densitometry screen window of the Casia. Left: Infrared image of the anterior segment; GSU values for cornea, anterior chamber and crystalline lens by means of the “Target Line” position (black arrow), the “Pupil Area”, and the “Optional Area” which has manually modifiable range of values. Center: OCT B scan image. Right: A scan graph of CD values throughout the anterior segment referring to the “Target Line” position

Four concentric zones were analyzed: the first was a round area measuring 2 mm in diameter (zone 1) and centered on the corneal apex, the other 3 zones were ring-shaped with a diameter ranging from 2 to 6 mm (zone 2), from 6 to 10 mm (zone 3), and from 10 to 12 mm (zone 4). The total corneal densitometry (TCD), referring to the measurement area comprised between 0 and 12 mm, was also evaluated. These topographical zones are set as default in the Pentacam software, whereas they need to be individually set in the Casia 2 software by clicking on “Settings” on the “DENSITOMETRY” page (Fig. 1). The 0.5–4 mm zone defined as “Pupil Area”, which is pre-set on the Casia 2, was used only for the evaluation of repeatability.

The output was expressed in gray scale units (GSUs) calibrated by each instrument’s software: in the case of the Pentacam AXL Wave, measurements were expressed as percent values ranged from 0% (minimum transparency) to 100% (maximum transparency), whilst for the Casia 2 they ranged from 0 GSU(minimum transparency) to 256 GSU(maximum transparency). Each measurement represents the CD measured from the epithelium to the endothelium. Densitometry values from each corneal zone are the mean of all the measurements of the respective zone.

### Statistical analysis

The statistical analyses were performed using SPSS (Version 25, IBM, United States). The sample size for the repeatability analysis was calculated to yield a minimum of 10% confidence in the estimate. According to McAlinden et al., at least 96 eyes should be enrolled for this purpose [23].

For the assessment of repeatability, as defined by the International Organization for Standardization, the following parameters were calculated [24]:

1. Intra-session test–retest variability, calculated by multiplying the pooled within-subject SD (sw) by 2.77. Based on repeatability, the difference between two measurements for the same subject should be less than 2.77 sw for 95% of pairs of observations [25].
2. CoV: calculated as the sw divided by the mean of the measurements and expressed as a percentage.
3. Intra-class correlation coefficient (ICC). This is defined as the ratio of the between-subjects variance to the sum of the pooled within-subject variance and the between-subjects variance [26, 27]. It was automatically calculated using SPSS software with the 2-way mixed model and absolute agreement. The range varies from 0 to 1, therefore, a value of 1.0 means no variance between repeated measurements, an ICC of 0.90 or more means high agreement, an ICC from 0.75 to less than 0.90 means moderate agreement, and an ICC of less than 0.75 means poor agreement [28].

To ensure comparability between the CD measurements of the two instruments, which are expressed in different scales (from 0 to 256 for Casia 2 and from 0 to 100 for Pentacam AXL Wave), Casia 2 densitometry results were converted to percentage, calculated as the densitometry results divided by the maximum value of densitometry readings for each instrument, multiplied by 100%. To detect differences in groups means or medians the t-test for paired samples was used for normally distributed variables; the non-parametric Wilcoxon signed-rank test was used for the variables that were not normally distributed. Pearson correlation analyses were used to quantify the linear correlation between the two sets of values obtained from the two devices. A *p*-value less than 0.05 was considered statistically significant.

To assess agreement, Bland and Altman plots were built with percentage values and with Z-score standardized values. To apply Z-score standardization, the mean of the correspondent sample is subtracted from each measurement (X) and then divided by the standard deviation of the sample according to the formula:

$$Z \,Score =\frac{X-mean}{standard \,deviation}$$

The resulting Z score distribution for each group of measurements has a mean of 0 and a standard deviation of 1. Each measurement is then expressed as the number of standard deviations from the mean of its sample.

Only the first measurement of each eye with both devices was used to evaluate agreement. This was assessed, according to Bland and Altman, by plotting the differences between measurements (y-axis) against their mean (x-axis) on the Z-scores [29]. The 95% limits of agreement (LoAs) were defined as the mean ± 1.96 standard deviation (SD) of the differences between the measurements of the two devices.

## Results

Ninety-six eyes from 96 healthy subjects were included in the study. The age ranged from 23 to 82 years and the mean was 42.28 years (mean ± SD = 42.37 ± 16.67 years). Fifty-four subjects were women and 44 were men.

### Repeatability

Table 1 summarizes the repeatability data obtained from the two instruments for each corneal zone. Overall, the Casia 2 revealed high repeatability for all measured parameters, as the CoV ranged from 1.07% to 2.25% and the ICC was consistently higher than 0.9 in all corneal zones. Compared to the Pentacam AXL Wave, the Casia 2 obtained lower CoV values in all corneal zones. According to the CoV, the variability of the measurements increased progressively from the center to the periphery, as the lowest score was observed in zone 1 and the highest in zone 4 for both instruments.

**Table 1:** Casia 2 and Pentacam AXL wave repeatability data

	Casia 2			Pentacam AXL wave		
	Test–retest repeatability (2.77sw; 95% CI)	Coefficient of variation (%; 95% CI)	ICC average measure (95% CI)	Test–retest repeatability (2.77sw; 95% CI)	Coefficient of variation (%; 95% CI)	ICC average measure (95% CI)
Zone 1	2.53 (1.95; 3.11)	1.07 (1.06; 1.07)	0.938 (0.901; 0.963)	0.77 (0.65; 0.95)	1.98 (1.95; 2.03)	0.976 (0.962; 0.986)
Zone 2	1.60 (1.13; 2.07)	1.17 (1.15; 1.18)	0.965 (0.945; 0.979)	0.60 (0.47; 0.71)	1.64 (1.60; 1.68)	0.988 (0.981; 0.993)
Zone 3	1.57 (0.98; 2.15)	1.39 (1.33; 1.46)	0.997 (0.996; 0.998)	1.27 (0.77; 1.85)	4.71 (4.31; 5.20)	0.994 (0.991; 0.997)
Zone 4	4.98 (4.04; 5.92)	2.25 (2.17; 2.33)	0.989 (0.983; 0.994)	3.64 (3.2; 4.59)	7.19 (6.63; 7.88)	0.981 (0.970; 0.989)
TCD	2.62 (1.97; 3.26)	1.61 (1.56; 1.66)	0.993 (0.989; 0.996)	1.02 (0.72; 1.32)	2.91 (2.76; 3.07)	0.992 (0.988; 0.995)
“Pupil area”	1.90 (1.31; 2.48)	1.16 (1.15; 1.17)	0.929 (0.886; 0.958)			

*sw* within-subject standard deviation, *ICC* intraclass correlation coefficient, *CI* confidence interval, *TCD* total corneal zone

### Values and agreement

Table 2 shows each measured parameter’s mean, SD, and 95% LoAs. The Casia 2 CD values ranged from 42.96 ± 1.13% in zone 1 to 26.19 ± 4.2% in zone 3 while the Pentacam AXL Wave’s ranged from 23.31 ± 6.68% in zone 4 to 15.84 ± 1.35% in zone 2. Casia 2 were found statistically significantly higher in each corneal zone (*p* < 0.0001). Pearson correlation revealed a tendency toward linear correlation in all the corneal zone except zone 1 (*p* = 0.35).

**Table 2:** Casia 2 and the Pentacam AXL wave CD value

	Casia 2 GSU mean ± SD (95% CI)	Casia 2 mean ± SD (%)	Pentacam mean ± SD (%)	*P* value	Pearson correlation *P* value	95% LoA (standardized values)	95% LoA (Bias) (%)
Zone 1	109.98 ± 2.90 (99.92–111.7)	42.96 ± 1.13 (42.64–43.29)	17.35 ± 1.33 (16.97–17.73)	< 0.0001*	0.35	± 2.42	22.38–28.95 (25.61)
Zone 2	71.45 ± 2.86 (67.29–72.92)	27.91 ± 1.12 (27.59–28.23)	15.84 ± 1.35 (15.46–16.23)	< 0.0001*	< 0.001	± 2.0	9,56–14,57 (12.07)
Zone 3	67.04 ± 10.76 (62.23–69.64)	26.19 ± 4.2 (24.98–27.40)	18.71 ± 6.04 (16.98–20.45)	< 0.0001†	< 0.0001	± 0.75	2.55–12.40 (7.48)
Zone 4	96.04 ± 12.37 (89.66–99.14)	37.52 ± 4.83 (36.13–38.90)	23.31 ± 6.68 (21.39–25.23)	< 0.0001†	< 0.0001	± 1.34	6.11–22.30 (14.21)
TCD	77.65 ± 9.02 (72.63–79.98)	30.33 ± 3.52 (29.32–31.35)	18.28 ± 3.31 (17.32–19.23)	< 0.0001†	< 0.0001	± 1.08	8.64–15.48 (12.06)

*SD* standard deviation, *LoA* limits of agreement,* Paired samples T-test, † Wilcoxon test

**Fig. 2:**
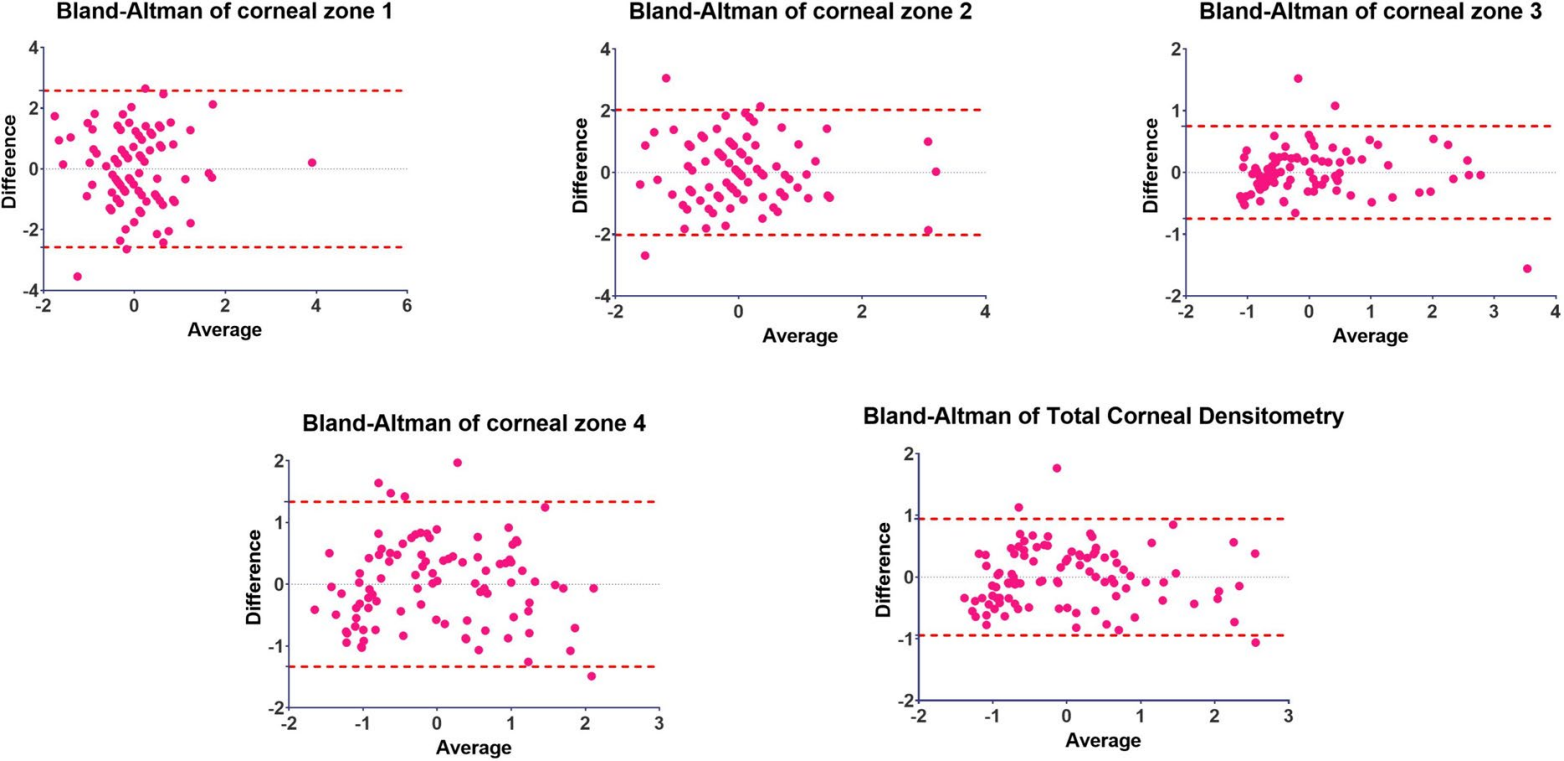
Bland–Altman plots for each corneal zone showing the average (x-axis) against the difference of each pair of measurements (*y*-axis). Central dotted line: Bias. Upper and lower dotted line: Limits of Agreement

The Bland and Altman analysis with percentage values shows a consistent bias in the average of the means for all zones Once Z-score standardization was applied (see Supplementary Material Fig. 1) the 95% LoA ranged from ± 2.42 for zone 1 and ± 0.75 for zone 3 (Table 2). Bland and Altman graphs are shown in Fig. 2.

### Correlation with age

**Fig. 3:**
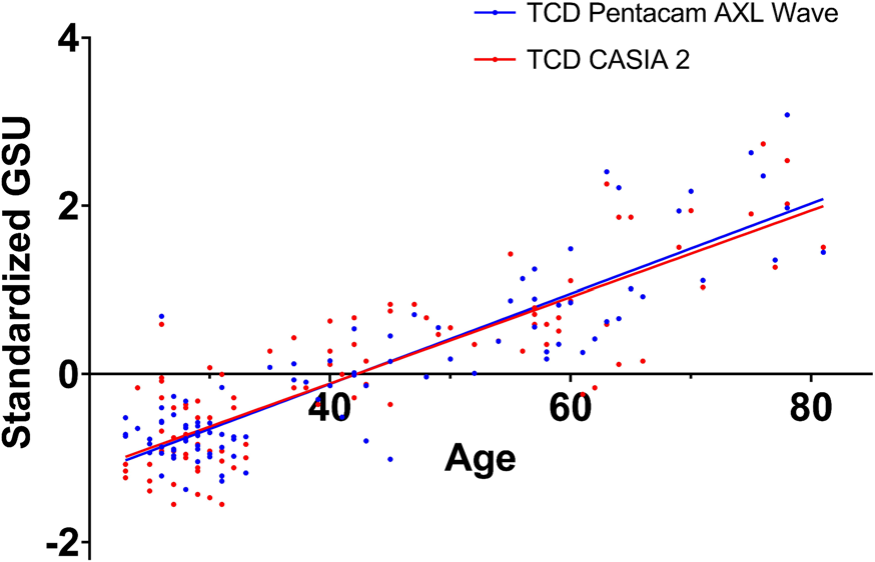
Linear regression between Age (x-axis) and Standardized GSU values (y-axis) for TCD. Both instruments demonstrate a positive relationship with age: Casia 2 r^2^: 0.76, *p*: < 0.0001. Pentacam AXL Wave r^2^: 0.8, *p*: < 0.0001. The difference between the two regression lines was examined using the extra sum of squares F-test, which confirmed that the distinction between the slopes is not statistically significant (*p* = 0.56). GSU: Gray Scale Unit. TCD: Total Corneal Densitometry

TCD showed a positive correlation with age for both instruments, as shown in Table 3 (Casia 2: *r* = 0.81, *p* < 0.001; Pentacam AXL Wave: *r* = 0.79, *p* < 0.001). The relationships between TCD and age is displayed in Fig. 3. There was a tendency towards a progressive increase in correlation with age proximal to the limbus up to the 10 mm diameter. The Pentacam measurements for zone 1 failed to show a significant correlation (*p* = 0.083) and in the case of the Casia 2 the same zone showed the lowest correlation among all the zones (*r* = 0.25 *p* = 0.0137); with both instruments the highest correlation was found in zone 3 (Casia 2: *r* = 0.82, *p* < 0.0001; Pentacam AXL Wave: *r* = 0.81, *p* < 0.0001).

**Table 3:** Correlation between CD, age, and CCT

	Age				CCT				Multiple regression analysis (*P* value < 0.025¥)	
	Casia 2		Pentacam AXL Wave		Casia 2		Pentacam AXL Wave		Age	CCT
	*p*	*r*	*p*	*r*	*p*	*r*	*p*	*r*	*p*	*p*
Zone 1	0.007*	0.25	NS*	–	0.02*	0.11	NS*	–	0.009	**NS**
Zone 2	< 0.0001*	0.53	< 0.0001*	0.49	NS*	–	NS*	–	–	–
Zone 3	< 0.0001†	0.82	< 0.0001†	0.81	**NS**†	–	**NS**†	–	–	–
Zone 4	< 0.0001†	0.71	< 0.0001†	0.75	0.018†	0.11	**NS**†	–	< 0.0001	**NS**
TCD	< 0.0001†	0.81	< 0.0001†	0.79	**NS**†	–	**NS**†	–	–	–

*CCT* central corneal thickness*NS* not significant (*p* > 0.05), ¥: Bonferroni corrected *P* value for multiple comparisons, *: Pearson correlation, †: Spearman correlation

### Correlation with gender

The CD was not correlated with gender in any corneal zone measured by the two instruments.

### Correlation with CCT

CD values showed a significant negative correlation in bivariate analysis for zones 1 and 4 as measured by the Casia 2 (*r* = -0.33, *p* = 0.02 and *r* = -0.34, *p* = 0.018) (Table 3). To account for the effect of CCT on densitometry, a multiple linear regression was performed using the Casia 2 CD values for zones 1 and 4. For this case, a Bonferroni adjusted significance threshold was applied, therefore, only a P-value less than 0.025 was considered statistically significant. After the correction for age, there was no significant influence of CCT on CD (Table 3).

## Discussion

This study aimed to evaluate the repeatability and agreement of CD measurements by an AS-OCT device, the Casia 2 and the Pentacam AXL Wave Scheimpflug camera in a group of healthy subjects with a minimal refractive error, and assess their correlation with age, CCT and gender. This was the first study to evaluate Casia 2 CD measurements repeatability, the AS-OCT device and the Scheimpflug camera system revealed high repeatability, with ICC values exceeding 0.9 in all the corneal zones. Previous reports have also demonstrated the high repeatability of the Pentacam CD measurements in normal eyes [9, 10, 30], in good agreement with our findings. When the repeatability of the two instruments was compared, the Pentacam consistently showed higher CoV values than the Casia 2 in all corneal zones, suggesting that the former provides lower repeatability compared to AS-OCT, with a larger difference towards the peripheral zones, as the Pentacam shows a sharper increase in CoV values in zones 3 and 4. Our results are in agreement with the ones provided by Ní Dhubhghaill et al., who warned against Pentacam CD measurements from the peripheral cornea, since they can be affected by scleral reflectivity and therefore demonstrate lower repeatability, especially if the corneal diameter is smaller than 12 mm [9].

**Fig. 4:**
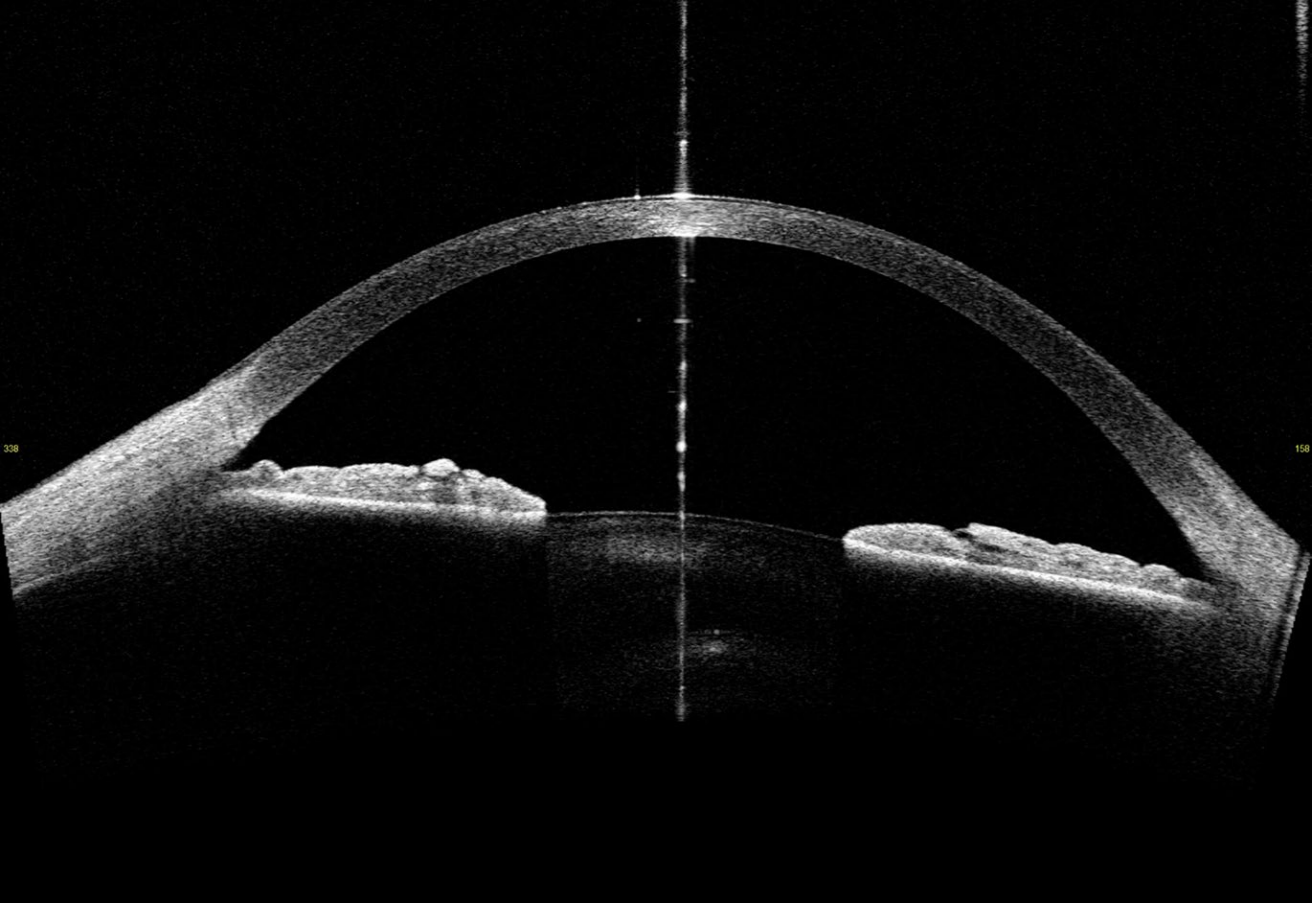
Casia 2 B scan image showing the central artifact line

Regarding agreement, the Bland–Altman plots showed a consistent bias through all the corneal zones, while, only, the 95% LoA of zone 1 were found to lie outside the range of ± 2 SD from the mean of the differences (± 2.6 SD for standardized values), indicating low agreement [29], moreover, it failed to show a statistically significant linear correlation. Despite the general goodcorrelation between measurements and narrow 95% LoA, CD measurements obtained from the two instruments exhibit differences in distribution and across the different corneal zones making their measurements not interchangeable. Specifically, CD values obtained with the Casia 2 were found to be higher in zone 1 compared to the other zones, while the Pentacam AXL Wave CD measurements were consistently higher in zone 4 and lower in zone 1, which is in line with previous literature findings in normal and myopic eyes (9, 22). These variations in CD measurements are likely attributable to the different technologies employed by the two instruments. The Pentacam AXL Wave utilizes a Scheimpflug camera system, which captures light backscattering with laterally positioned cameras while the emitting light surce is centered on the visual axis. The Casia 2, by contrast, registers reflected light along the same axis as the one of light emission, leading to enhanced measurements in the central zones where the light has a reflection angle close to zero and attenuated measurements in the peripheral zones. Notably, the central artefact line **(**Fig. 4**)** observed in the Casia 2 images represents the axis where the light strikes the corneal apex perpendicularly, resulting in a strong registration signal that may appear as an artefact. To address this issue, the Casia 2 software excludes the central 0.5 mm where this artefact line appears in the densitometry analysis.

Our study aimed to analyze eyes with small refractive error to minimize the effect of highly ametropic eyes on CD [31]. Nevertheless, a recent study by Xu et al. [22] analyzed CD values between Pentacam and Casia 2 in myopic eyes including refractive errors higher than -6D. Compared to our study, their results revealed similar differences of distributions across the four corneal zones for both instruments and lower agreement in the central zone, albeit with no statistical significance. Notably, their study included subjects within the age range of 18–41 years. In contrast, our study encompassed participants aged 23–82 years. Consequently, their findings consistently revealed lower CD values compared to those collected in our study and can not be further compared.

Despite the lack of interchangeability of measurements between the devices due to their distinct imaging principles, both data sets demonstrated comparable trends and exhibited a significant positive linear relationship in most zones. Therefore, it can be said that Casia 2 can detect densitometry changes in healthy eyes with limited refractive error, comparably with the Pentacam, peripherally to the 2 mm from the corneal apex. Further investigation is warranted to determine whether the differences exhibited in zone 1 could affect the clinical value of densitometry measurements, and whether the exclusion of the central artifact line could potentially impact the agreement between the two instruments in the central corneal zone. While it is unlikely to significantly affect the reliability of CD measurements in healthy and uniform corneas, its potential impact on CD measurements in pathological corneas, such as those with keratitis, keratoconus, or Fuchs dystrophy, needs to be evaluated.

Our study revealed that CD was positively correlated with age using both instruments in all corneal zones, except for zone 1, where the Pentacam AXL Wave did not show a statistically significant correlation. This finding could be attributable to age-related alterations that occur in each corneal layer. These changes may include a decrease in keratocyte and endothelial cell density, impairment of the epithelial cell barrier function, alteration of collagen fibers, and the presence of cellular debris within the collagen lamellae [32]. However, as regards the relationship between central CD and age, conflicting results are to be found in the literature. Previous studies have reported no correlation between CD and age in central corneal zones measured with a Scheimpflug camera [4, 6, 8, 9], as well as with other methods [33–35]. In contrast, Karmiris et al. [36] reported a significant correlation between age and 0–2 mm CD in each corneal layer and throughout the total thickness using the Pentacam. However, the study is limited by the inclusion of both eyes of the same subject. Based on our study and the literature findings, it can be postulated that the Pentacam AXL Wave may lack the resolution required to detect subtle age-related changes in CD within the central corneal zone. The AS-OCT device may be more sensitive in this regard. On the other hand, there is a consensus in the literature regarding the effect of corneal aging in the more peripheral zones and this effect increases as one proceeds toward the limbus [7, 8]. This may account for the more pronounced involvement of the peripheral cornea in involutional changes such as arcus senilis and Vogt’s girdle [32].

In agreement with the literature, densitometry values did not show a correlation with CCT [8], nor was there any difference between genders [9].

Comparing two instruments with different mechanisms poses some inconveniences, especially when differences between subjects are less obvious, as it is in healthy subjects with clear corneas, the instrument’s capacity is put under a stress and small technical differences between protocols and environmental factors might play a role in defining repeated measurements. In our case, images were obtained in the same room and light conditions for each patient, and in random instrument order, to eliminate external influences.

This study has some limitations and further investigation is warranted, enrolling only healthy eyes with minimal refractive error prevents our results from being generalized to patients with any corneal disease or more pronounced refractive error. It was not possible to compare CDs from different corneal layers, since the Casia 2 software does not provide such information.

In conclusion, our data indicate high repeatability of CD measurements obtained by the Casia 2. However, due to the differing principles of image capturing, the absolute values were not interchangeable with those obtained by the Pentacam. Additionally, The Casia 2 revealed more sensibility to age-related changes in the central zone. Further studies are needed to confirm this finding and evaluate the potential clinical implication for understanding and managing corneal health and pathologies.

## Supplementary Information

Below is the link to the electronic supplementary material.

Supplementary file1 (DOCX 171 KB)

## Data Availability

No datasets were generated or analysed during the current study.
