# Supplementary material for: Corneal densitometry measurements comparison between anterior segment OCT and scheimpflug imaging
Source: Int Ophthalmol. 2024 Sep 25;44(1):392. doi: 10.1007/s10792-024-03309-0 (PMC11424699; doi:10.1007/s10792-024-03309-0)

# Supplementary material

Supplementary Material Figure 1: *Histogram of Z-score standardization distribution in each corneal zone*


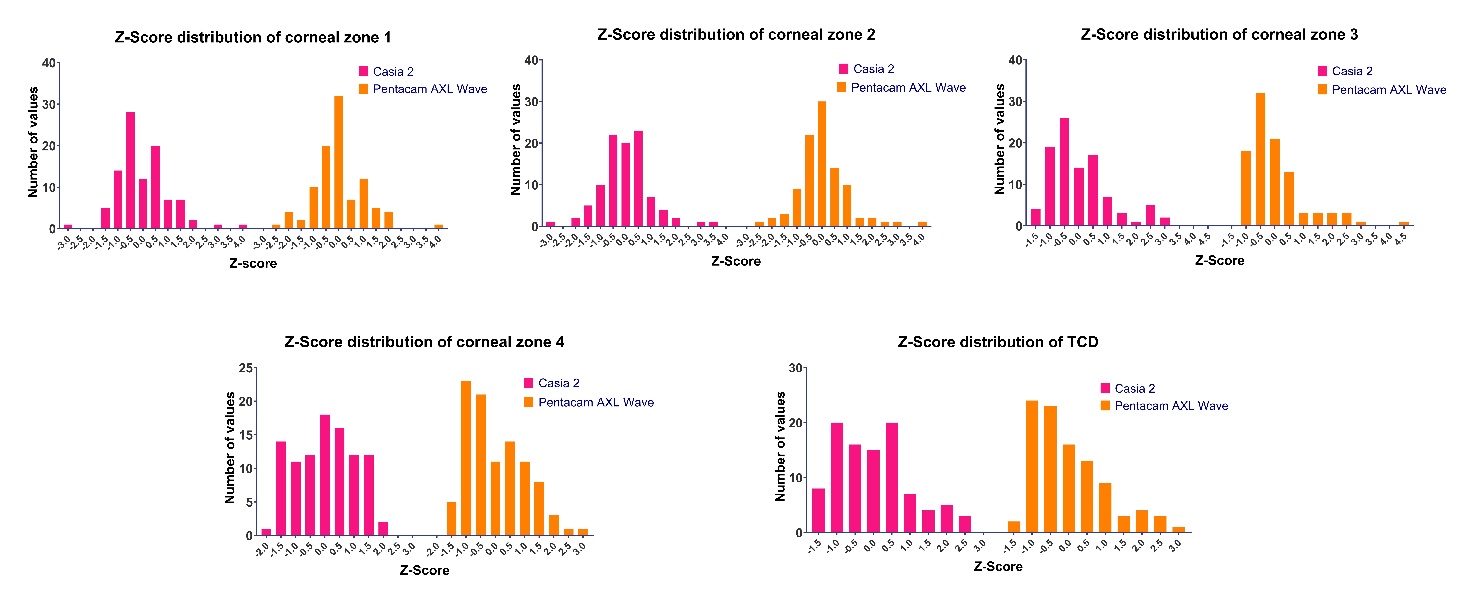

Supplement: Supplementary file 1 — Supplementary file1 (DOCX 171 KB) [file 10792_2024_3309_MOESM1_ESM.docx]
